# Bart’s Syndrome Associated Corpus Callosum Agenesis and Choanal Atresia

**Published:** 2014

**Authors:** Muhammad SAEED, Anwar ul HAQ, Khaqan QADIR

**Affiliations:** 1 King Faisal Specialist Hospital & Research Center, Riyadh, Saudi Arabia; 2 Military Hospital Riyadh, Riyadh, Saudi Arabia

**Keywords:** Bart’s syndrome, Congenital absence of skin, Epidermolysis bullosa, Choanal atresia, Corpus callosum agenesis (CCA)

## Abstract

Bart’s syndrome is defined as congenital localized absence of skin, and associated with epidermolysis bullosa. A newborn with Bart’s syndrome is reported because it is a very rare condition, especially when associated with corpus callosum agenesis and concomitant choanal atresia. Clinically it is characterized by raw beefy areas of denuded skin mainly on hands and feet.

We report a rare case of a term female newborn born to non-consanguineous parents who presented with congenital absence of skin in, face, trunk and extremities. To the best of our knowledge, this is the first report presenting a case of Bart’s syndrome associated with corpus callosum agenesis.

## Introduction

Bart’s syndrome is clinically described as the association of congenital localized absence of skin (CLAS), epidermolysis bullosa (EB), and nail abnormalities. Bart’s syndrome, first described by Bart in 1966, consists of congenital localized absence of skin, congenital epidermolysis bullosa, and associated nail abnormalities (1).

This association is described in the literature as Bart Syndrome (2). 

We describe here a case of newborn baby who presented with denuded skin on the neck, knees, hands and feet. Clinical findings were sufficiently supportive to suggest the diagnosis of Bart’s syndrome. We repot this case because of its rarity with corpus callosum agenesis.

## Case Report

A female newborn born by normal vaginal delivery at the 36th week of gestation admitted neonatal unit of Jinnah Hospital Lahore, she was noted to have absence of skin on lateral sites of her neck, lower part of face, legs above the ankle, on the both knees, as well as the dorsum of both hands and feet. She was the second child of a non-consanguineous couple.

**Fig 1:**
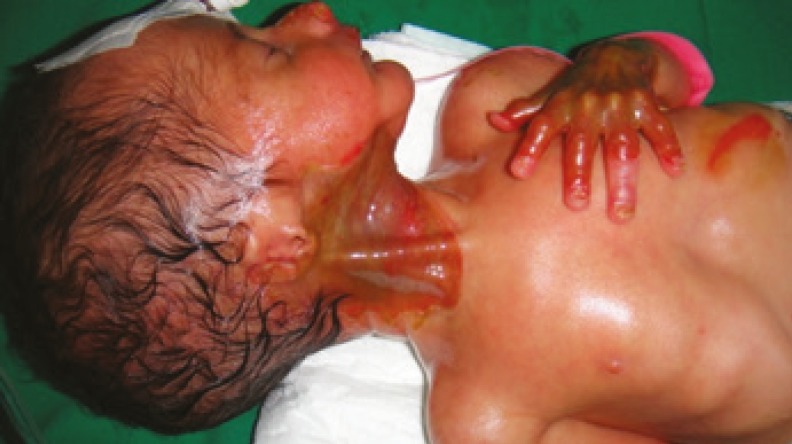
Absent skin, on tip of nose, neck, bilaterally on the upper and lower extremities

**Fig 2:**
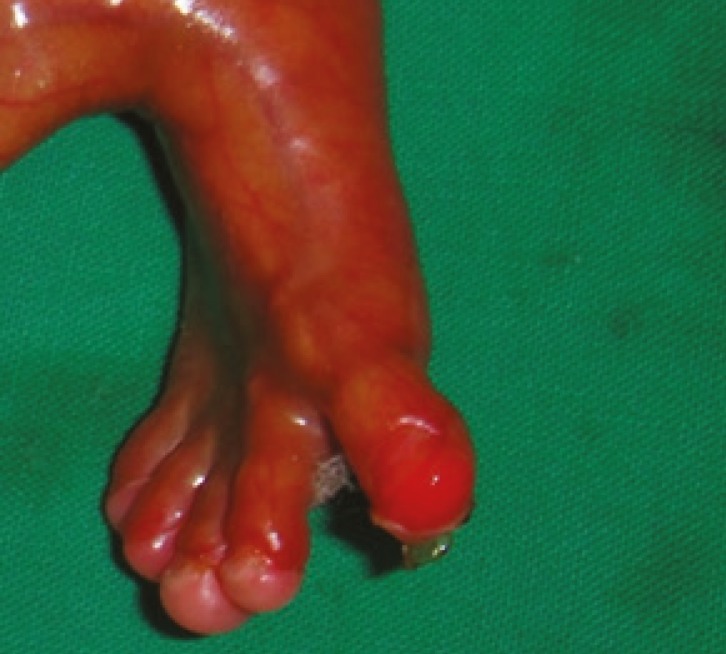
Absent Skin on Foot and Toes

**Fig 3.**
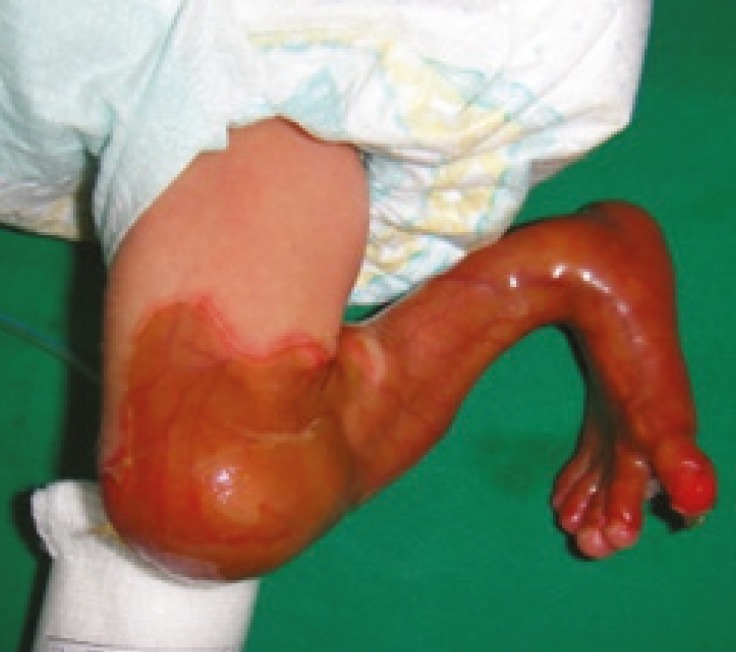
Absent Skin on Knee Joint, Leg and Foot

**Fig 4:**
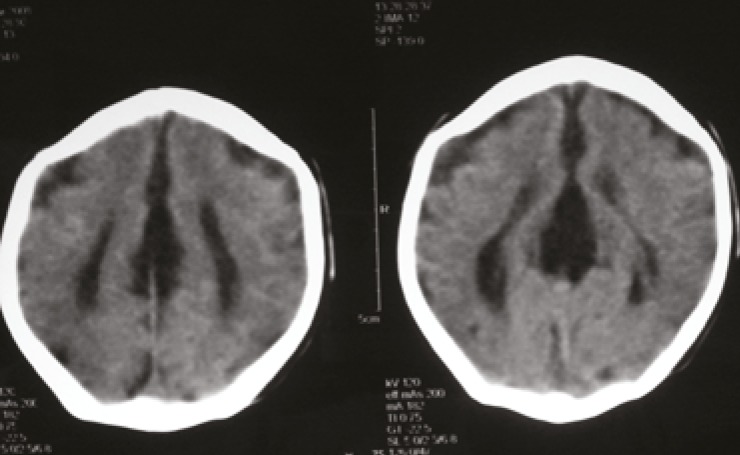
CT Brain, showing Agenesis of Corpus Callosum

In addition to the absence of skin, she had hypoplastic nails (Figures 1-4).

The mother had no specific past medical history. The birth weight, length and head circumference were 1600 g (<3rd percentile), 39 cm (<3rd percentile), and 33 cm (50th percentile), respectively. 

We tried pass the feeding tube from the right nostril but could not succeed. All routine investigations and TMS were normal. Echocardiography and abdominal ultrasound examinations were normal. On cranial ultrasonography it was reported absence of corpus callosum, so we performed CT brain which confirmed the absent of Corpus callosum. Cytogenetic study of the patient showed a normal female karyotype 46 XX and no chromosomal abnormality was detected. 

Thus the diagnosis of Bart’s syndrome with epidermolysis bullosa dystrophica was made. We could not carry out ultrastructural, immunohistologic and genetic linkage studies because of nonavailability.

The patient was diagnosed as having congenital choanal atresia and corpus callosum agenesis associated with Bart’s syndrome. However, no other congenital abnormality detected. On the tenth day of admission after her birth, she died due to septicemia.

## Discussion

Bart’s syndrome was first reported in 1966 in a family with congenital localized absence of skin (CLAS) on the lower leg, widespread blistering of the skin and mucous membranes and nails dystrophy (1). This unique association came to be known after his name as Bart’s syndrome. Bart considered the congenital absence of skin as an occasional manifestation of Epidermolysis bullosa and attributed it to in utero blistering (2).

Epidermolysis bullosa-CLAS cases were reported in the literature. The pathogenesis of aplasia cutis congenital (ACC), in the setting of EB, is unknown (3,4). Keeping this view Kanzler et al. suggested abandoning Bart’s syndrome as separate disease entity (5) Mechanical trauma could occur from fetal movements, such as rubbing, leading to in utero blistering with subsequent erosions (6).

The presentation of dystrophic epidermolysis bullosa with congenital localized absence of skin (Bart’s Syndrome) is rare entity, with only few cases described in the published literature (7). The condition may be associated with epidermolysis bullosa, specific teratogens or intrauterine infection, or it may occur in the presence of Chromosomal abnormalities (8). 

However, Bart could not properly classify the disease as ultrastructural and Immunochemical studies were not available at that time. Later Zelickson et al. carried out these studies on the original kindred described by Bart and proved that these were cases of dominant dystrophic EB associated with congenital absence of skin. Subsequently Joensenin in 1973 and Skoven and zewiecki in 1979 reported similar cases (9,10).

Most commonly, the limbs and extremities and also sometimes the parietal and occipital region of the scalp are affected in CLAS (11). 

Symmetric distribution, sharply demarcated borders, and involvement of toe webs and soles are frequent (12). 

The syndrome is inherited in autosomal dominant fashion, apparently with full penetrance but with variable expressivity. The disease results from a substitution within the type VII collagen gene (13,14). Multiple congenital anomalies of the other systems, are associated with Barts syndrome. To the best of our knowledge, this is the first report presenting a case of Bart’s syndrome associated with corpus callosum agenesis. 

In newborns with congenital absence of the skin in the presence of bullae formation, Bart’s syndrome should be considered, and the newborns should be examined in terms of developmental defects of other systems including CNS. Genetic counseling for affected families and sonographic follow-up in order to find evidence of upper intestinal obstruction in the prenatal period is extremely important for diagnosis of this rare familial disorder.

**In conclusion, **treatment of Barts syndrome is conservative for most patients, especially for those with cerebral anomalies. Plastic repair has been recommended in certain instances. In spite of adequate supportive and appropriate antibiotic therapy, our patient died. We think that the neurological disorders including microcephaly, CCA are part of syndrome, so any case of Bart’s syndrome must be evaluated for cerebral anomalies. This case report may help future researchers on the subject in elucidating the causation of corpus callosum agenesis in Bart’s syndrome.

## References

[1] Kharkar V, Pande S, Mahajan S, Dwiwedi R, Khopkar U (2007 1). Griscelli syndrome: a new phenotype with circumscribed pigment loss?. Dermatol Online J.

[2] Sheela SR, Latha M, Susy JI (2004). Griscelli syndrome: Rab 27a mutation. Indian Pediatrics.

[3] González Carretero P, Noguera Julian A, Ricart Campos S, Fortuny Guasch C, Martorell Sampol L (2009). Griscelli-Prunieras syndrome: report of two cases. An Pediatr (Barc).

[4] Szczawinska-Poplonyk A, Kycler Z, Breborowicz A, Klaudel-Dreszler M, Pac M, Zegadlo-Mylik M (2011 Dec). Pulmonary lymphomatoid granulomatosis in Griscelli syndrome type 2. Viral Immunol.

[5] Durmaz A, Ozkinay F, Onay H, Tombuloglu M, Atay A, Gursel O (2012). Molecular analysis and clinical findings of Griscelli syndrome patients. J Pediatr Hematol Oncol.

[6] Reddy RR, Babu BM, Venkateshwaramma B, Hymavathi Ch (2011). Silvery hair syndrome in two cousins: Chediak-Higashi syndrome vs Griscelli syndrome, with rare associations. Int J Trichology.

[7] Sahana M, Sacchidanand S, Hiremagalore R, Asha G (2012). Silvery grey hair: clue to diagnose immunodeficiency. Int J Trichology.

[8] Mahalingashetti PB, Krishnappa MH, Kalyan PS, Subramanian RA, Padhy S (2012). Griscelli syndrome: hemophagocytic lymphohistiocytosis with silvery hair. J Lab Physicians.

[9] Schuster F, Stachel DK, Schmid I, Baumeister FA, Graubner UB, Weiss M (2001). Griscelli syndrome: report of the first peripheral blood stem cell transplant and the role of mutations in the RAB27A gene as an indication for BMT. Bone Marrow Transplant.

[10] Shamsian BS, Nikoufar M, Esfahani SA, Shamshiri AR, Arzanian MT, Alavi S (2011). A 10-year single center survey of pediatric patients with histiocytic disorders in Iran. Turk J Pediatr.

[11] Birnbaum RY, Landau D, Elbedour K, Ofir R, Birk OS, Carmi R (2008). Deletion of the first pair of fibronectin type III repeats of the integrin beta-4 gene is associated with epidermolysis bullosa, pyloric atresia and aplasia cutis congenita in the original Carmi syndrome patients. Am J Med Genet.

[12] Rajpal A, Mishra R, Hajirnis K, Shah M, Nagpur N (2008). Bart’s syndrome. Indian J Dermatol.

[13] Zelickson B, Matsumara K, Kist D, Epstein EH Jr, Bart BJ (1995). Bart’s syndrome: Ultrastructure and genetic linkage. Arch Dermatol.

[14] Christinano AM, Bart BJ, Epstein EH Jr, Uitto J (1996). Genetic basis of Bart’s syndrome: A glycine substitution mutation in the type VII collagen gene. Invest Dermatol.

